# Endoscopic clearance of non‐complex biliary stones using fluoroscopy‐free direct solitary cholangioscopy: Initial multicenter experience

**DOI:** 10.1002/deo2.241

**Published:** 2023-06-01

**Authors:** Wiriyaporn Ridtitid, Rungsun Rerknimitr, Mohan Ramchandani, Sundeep Lakhtakia, Raj J Shah, Janak N Shah, Nirav Thosani, Mahesh K Goenka, Guido Costamagna, Mihir S Wagh, Vincenzo Perri, Joyce Peetermans, Pooja G Goswamy, Zoe Liu, Srey Yin, Subhas Banerjee

**Affiliations:** ^1^ Department of Medicine, Faculty of Medicine Chulalongkorn University and King Chulalongkorn Memorial Hospital Thai Red Cross Society Bangkok Thailand; ^2^ Asian Institute of Gastroenterology Hyderabad India; ^3^ Division of Gastroenterology and Hepatology University of Colorado Hospital Aurora USA; ^4^ Division of Gastroenterology Ochsner Clinic Foundation New Orleans USA; ^5^ Ertan Digestive Disease Center‐Texas Medical Center Houston USA; ^6^ Institute of Gastrosciences and Liver Apollo Multispeciality Hospitals Kolkata India; ^7^ Digestive Endoscopy Unit Department of Translational Medicine and Surgery Fondazione Policlinico Universitario Agostino Gemelli IRCCS, Università Cattolica del Sacro Cuore Rome Italy; ^8^ Boston Scientific Corporation Marlborough USA; ^9^ Division of Gastroenterology and Hepatology Stanford University Stanford USA

**Keywords:** bile duct stone, cholangioscopy, endoscopic retrograde cholangiography, fluoroscopy‐free, gastrointestinal endoscopy

## Abstract

**Background and Aims:**

Fluoroscopy‐free endoscopic retrograde cholangiopancreatography for common bile duct stone (CBDS) clearance is usually offered only to pregnant patients. We initiated a multicenter, randomized controlled trial comparing clearance of non‐complex CBDSs using fluoroscopy‐free direct solitary cholangioscopy (DSC) to standard endoscopic retrograde cholangiography (ERC) to evaluate the wider applicability of the DSC‐based approach. Here we report the initial results of stone clearance and safety in roll‐in cases for the randomized controlled trial.

**Methods:**

Twelve expert endoscopists at tertiary care centers in four countries prospectively enrolled 47 patients with non‐complex CBDSs for DSC‐assisted CBDS removal in an index procedure including fluoroscopy‐free cannulation. Successful CBDS clearance was first determined by DSC and subsequently validated by final occlusion cholangiogram as the ERC gold standard.

**Results:**

Fully fluoroscopy‐free cannulation was successful in 42/47 (89.4%) patients. Brief fluoroscopy with minimal contrast injection was used in 4/47 (8.5%) patients during cannulation. Cannulation failed in 1/47 (2.1%) patients. Fluoroscopy‐free complete stone clearance was reached in 38/46 (82.6%) cases. Residual stones were detected in the validation ERC occlusion cholangiogram in three cases. Overall serious adverse event rate was 2.1% (95% confidence interval 0.1–11.3): postprocedural pancreatitis in one patient.

**Conclusions:**

In patients with non‐complex CBDS, the fluoroscopy‐free technique is easily transferred to endoscopic retrograde cholangiopancreatography experts with acceptable rates of cannulation and stone clearance and few serious adverse events. (ClinicalTrials.gov number, NCT03421340)

## INTRODUCTION

Endoscopic retrograde cholangiopancreatography (ERCP), the standard endoscopic technique to manage common bile duct stones (CBDS), is performed with fluoroscopic guidance.^1^, ^2^, ^3^ Personnel involved in ERCP are at risk for cumulative radiation accumulation injury.^4^, ^5^ The American Society for Gastrointestinal Endoscopy and European Society of Gastrointestinal Endoscopy recommend using radiation doses as low as reasonably achievable (ALARA principle).^4^, ^5^, ^6^ Because clearance of non‐complex CBDS is the most common indication for ERCP, avoiding or reducing associated radiation exposure is desirable. In addition, not all patients are candidates for fluoroscopy‐guided ERCP, including gravely ill patients receiving intensive care who cannot be transferred to the fluoroscopy unit,^7^, ^8^ and patients during early pregnancy.^9^, ^10^, ^11^, ^12^ Because it can be performed at the bedside, direct solitary cholangioscopy (DSC) may be the only option to remove CBDS in intensive care unit patients.^13^ Bedside fluoroscopy‐free stenting is technically challenging including for selection of appropriate stent length and controlling the position of the guidewire tip, unless DSC is used.^7^, ^8^

Recently, some techniques to perform CBDS clearance without fluoroscopy have emerged including transabdominal ultrasound (TUS), endoscopic ultrasound (EUS)‐facilitated stone extraction, and DSC.^7^, ^13^, ^14^, ^15^, ^16^, ^17^, ^18^ Fluoroscopy‐free CBDS removal with DSC‐based stone clearance confirmed using a cholangioscope can be performed through the duodenoscope,^13^, ^18^ whereas a EUS‐facilitated approach requires one or more endoscope exchanges and TUS requires a change of the patient's positioning to evaluate for CBDS clearance.^7^, ^14^, ^15^, ^16^ To date, only two prospective single‐center studies reported the feasibility and safety of cholangioscopy‐based management of uncomplicated CBDS.^13^, ^18^ Therefore, a protocol‐based approach to cholangioscopy‐guided CBDS clearance warrants confirmation of applicability in a multicenter study.

## MATERIALS AND METHODS

We initiated a multicenter, randomized controlled trial comparing clearance of non‐complex CBDSs using fluoroscopy‐free DSC to standard ERCP to evaluate the wider applicability of the DSC‐based approach. Prior to enrolling in the randomized controlled trial, each investigator completed up to five roll‐in cases to ensure familiarity with the DSC technique.

### Study design and patient population

These roll‐in cases were conducted by 12 experienced endoscopists at eight tertiary referral centers in India, Italy, Thailand, and the USA. Participating endoscopists were highly experienced in ERCP (lifetime experience > 2000 cases) and in endoscopic cholangiopancreatoscopy (lifetime experience >200 cases). Consecutively enrolled patients met the following criteria: 18 years or older, abdominal pain consistent with CBDS, abnormal liver function tests, non‐complex biliary stone disease (See Supporting Information for *Definitions*) seen on prior recent non‐invasive imaging. If the probability of CBDS was low or intermediate according to American Society for Gastrointestinal Endoscopy criteria^19^ either magnetic resonance cholangiopancreatography or EUS imaging was necessary to confirm the presence of CBDS, whereas TUS was sufficient in those with a high probability of CBDS. All participating patients provided written informed consent. (See Supporting Information for exclusion criteria.)

Roll‐in cases were performed using protocolized procedural steps. Study data were recorded on standardized case report forms in a password‐protected electronic data capture system. All centers obtained study protocol and informed consent form approved by their local institutional review board. The study was registered on ClinicalTrials.gov (NCT03421340).

### Fluoroscopy‐free direct solitary cholangioscopy procedure

#### Fluoroscopy‐free cannulation

See Supporting Information.

### Cholangioscopy to confirm the number and estimated size of CBDS

**FIGURE 1:**
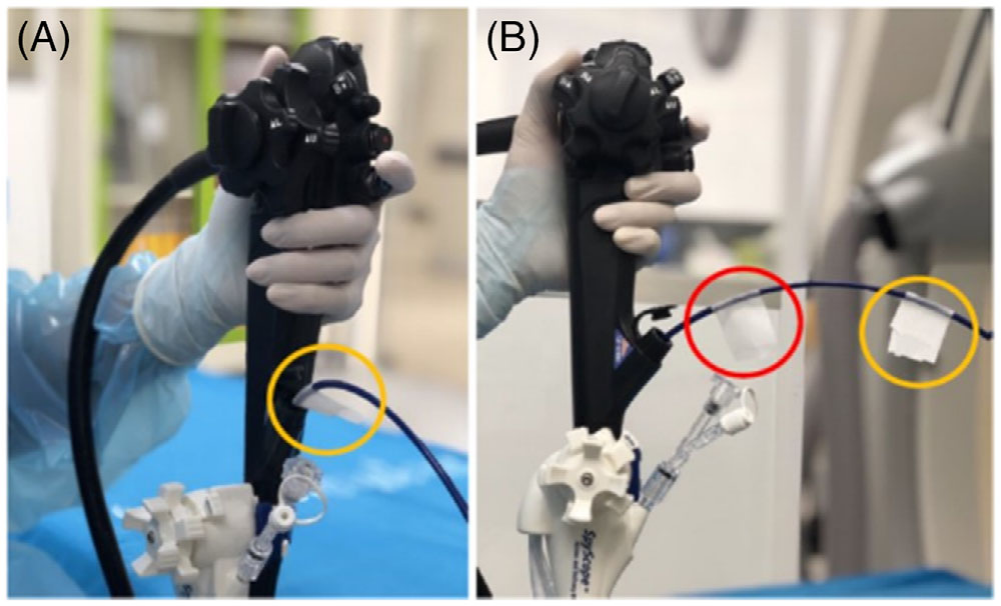
Distance of common bile duct between the hilum and the ampulla which was measured using adhesive tape placed on the cholangioscope shaft; (a) The first tape (yellow circle) was placed on the cholangioscope shaft where it exited the duodenoscope working channel when cholangioscope was at the hilum. (b) The second tape (red circle) was placed again more distally when it just exited the ampulla.

Following biliary sphincterotomy, the sphincterotome was exchanged to the direct solitary cholangioscope (SpyGlass DS Direct Visualization System; Boston Scientific, Marlborough, MA, USA) over the guidewire. The cholangioscope was advanced into the bile duct and the number and size of CBDSs were recorded (Video 1). Correct placement of the guidewire in the bile duct (and not in the cystic duct) was confirmed. Injection of saline/water was minimized to avoid driving stones into the intrahepatic ducts. Suction was applied through the cholangioscope as necessary. The distance from bifurcation to ampulla was measured using adhesive tape placed on the cholangioscope shaft where it exited the duodenoscope working channel when the cholangioscope was positioned at each of these locations (Figure 1).

**VIDEO 1:** The index procedure of patients undergoing direct solitary cholangioscopy‐assisted stone removal.

### Fluoroscopy‐free stone removal

All visualized CBDSs were extracted using a balloon catheter and/or SpyGlass Retrieval Basket. The balloon size was selected based on the common duct diameter determined by prior non‐invasive imaging. A stone extraction balloon catheter was marked with the distance from the ampulla to the hilum and then advanced over the guidewire. For a patient with multiple stones, the balloon was sequentially advanced more proximally step‐by‐step to avoid sweeping too many stones at once and eventually advanced to the hilum guided by the previously measured depth of insertion. Where only a single stone was noted, a single sweep commencing at the hilum was performed (Video 1). Alternatively, CBDSs were captured and retrieved sequentially using the SpyGlass Retrieval Basket under cholangioscopic visualization. If stone extraction by balloon sweep or basket capture was deemed difficult due to stone size, then balloon papillary dilation or DSC‐guided electro‐hydraulic lithotripsy (EHL) or laser lithotripsy could be performed before another attempt at CBDS extraction. If complete CBDS clearance still failed despite all the techniques described, plastic stent placement was allowed for temporary drainage, and the patient was rescheduled for a standard‐of‐care endoscopic retrograde cholangiography (ERC).

### Cholangioscopy to confirm complete stone clearance

Following successful stone removal, the cholangioscope was reinserted and advanced to the hilum to confirm complete stone clearance (Video 1). If residual stones were noted, additional cycle/s of balloon sweeps or basket capture followed by repeat cholangioscopy to confirm CBDS clearance were performed as necessary.

### Validation procedure

To validate DSC‐guided stone clearance, an occlusion cholangiogram was performed, and the presence or absence of filling defects was recorded. If filling defects were noted, balloon sweeps of the bile duct were performed to determine if the filling defects were “missed” CBDSs. The size of the largest missed stone and the number of “missed stones” >5mm per endoscopist's estimate were documented.

## 24‐h, 7‐DAY, AND 30‐DAY FOLLOW‐UP

Patients were followed at 24‐h, 7‐day, and 30‐day post‐index procedures. All adverse events such as cholangitis, pancreatitis, perforation, bleeding, and any other device or procedure‐related events were recorded. If the last follow‐up visit was not completed, the reason was reported.

### Definitions

See Supporting Information.

### Endpoints

The primary endpoint was complete stone clearance by extraction of bile duct stones from the common bile duct (CBD) into the duodenum as determined by fluoroscopy‐free cholangioscopy in the DSC arm and by cholangiography in the ERC arm.

Secondary endpoints included: 1) biliary cannulation success at the index DSC procedure, 2) stone removal at the index DSC procedure, 3) ERC validation of stone clearance after DSC, 4) operator rating of image quality, 5) duration of the index procedure, 6) radiation exposure to the patient, and 7) serious adverse events (SAEs) through 30 days post‐procedure. (See Supporting Information for details.)

### Statistical analysis

We reported baseline characteristics, and procedural and postprocedure data using descriptive statistics (mean ± standard deviation, or median, range) for continuous variables. Categorical variables were reported as numbers and percentages.

## RESULTS

### Patients

We enrolled 47 roll‐in patients (19 males, mean age 53.4 ± 18.5 years). Median time since the onset of abdominal pain was 48.0 (range 6–600) hours. Four (4/47, 8.5%) patients experienced acute pancreatitis in the 4 weeks prior to enrollment. Ten (10/47, 21.3%) patients had a prior cholecystectomy. Imaging suggesting CBDS included TUS in 16/47 (34.0%), abdominal CT scan in 13/47 (27.7%), magnetic resonance cholangiopancreatography in 15/47 (31.9%), and EUS in 15/47 (31.9%). Based on these imaging studies, the median diameter of the bile duct was 9.0 (range 3.0–17.0) mm and the median number of CBDS was 1.0 (range 0.0–5.0).

### Biliary cannulation at index DSC procedure

**FIGURE 2:**
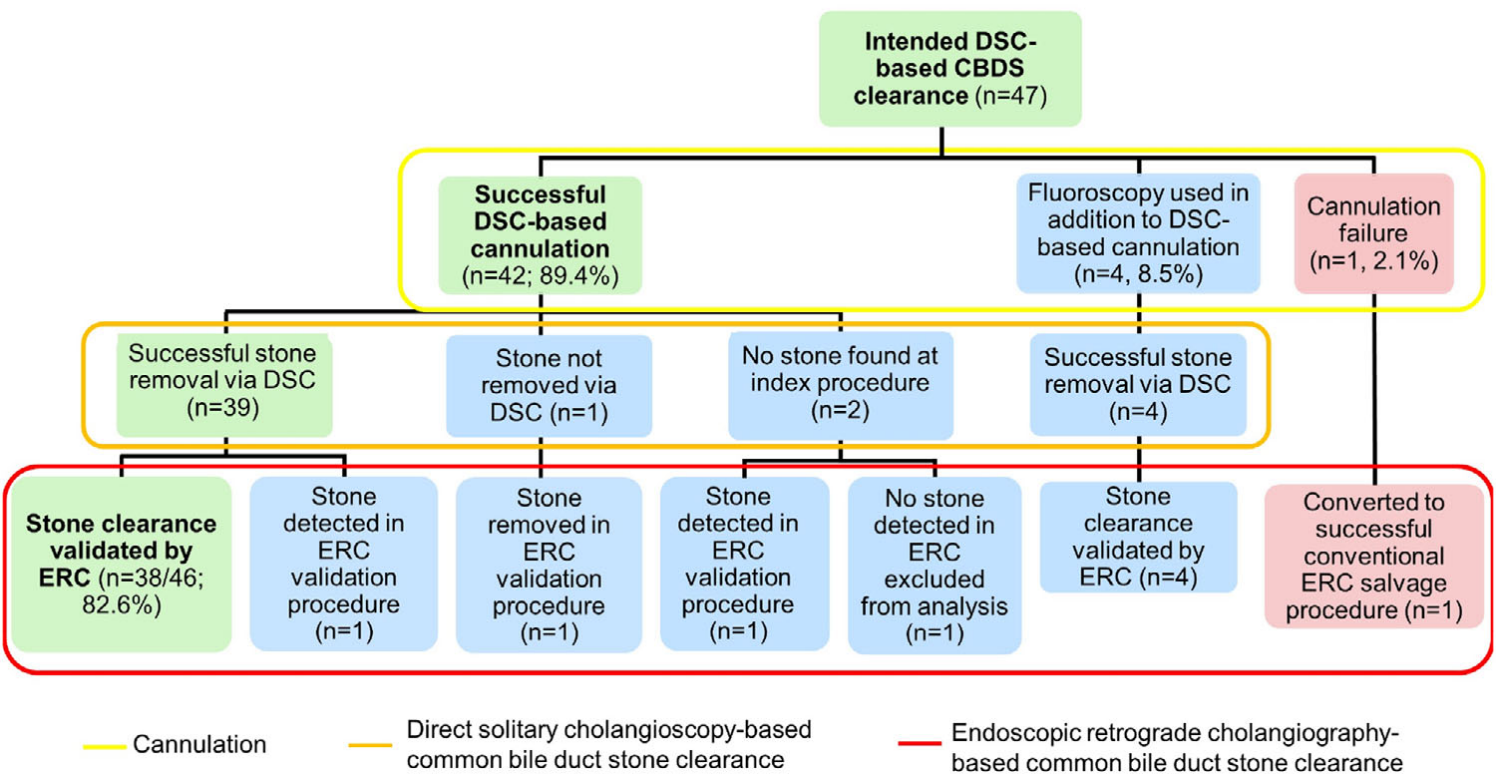
Flowchart of roll‐in patients undergoing direct solitary cholangioscopy‐assisted stone removal. CBDS, common bile duct stones; DSC, direct solitary cholangioscopy; ERC, endoscopic retrograde cholangiography.

Biliary cannulation without fluoroscopy was achieved in 42 (42/47, 89.4%) patients. Of these 42 cases, all but one underwent cannulation via standard technique, and one required needle knife assistance for a visibly impacted stone. In 5 patients in whom fluoroscopy‐free cannulation failed, one required advanced cannulation techniques including double guidewire and trans‐pancreatic septotomy, and failed in the index DSC procedure, but cannulation was achieved in the ERC salvage procedure immediately following DSC. In the other four patients, brief fluoroscopy was used to confirm DSC‐guided cannulation, followed by successful DSC‐guided CBDS clearance in all cases, using standard cannulation (1) or advanced (3) techniques (Table 1 and Figure 2). Inadvertent pancreatic duct cannulation was observed in 10/47 (21.3%), in cases with purely fluoroscopy‐free cannulation (6), with the use of a small amount of contrast injection and brief fluoroscopy (3), or with biliary cannulation failure (1).

**TABLE 1:** Stone removal using direct solitary cholangioscopy and validation using endoscopic retrograde cholangiopancreatography (*n* = 47)

Procedure	*N* (%)
Biliary cannulation (*n* = 47)	
Cannulation techniques	
Direct standard biliary cannulation without inadvertent pancreatic duct cannulation	36/47 (76.6)
Subsequent standard biliary cannulation after one or more pancreatic duct cannulation	10/47 (21.3)
Biliary cannulation with advanced techniques	4/47 (8.5)
Successful biliary cannulation	46/47 (97.9)
The use of fluoroscopy	
Fluoroscopy‐free cannulation	42/47 (89.4)
Brief fluoroscopy used in cannulation	4/47 (8.5)
Cannulation failure	1/47 (2.1)
Stone removal detail (*n* = 45)*	
Papillary balloon dilation	13/45 (28.9)
Methods of successful stone removal (*n* = 44)	
Balloon extraction	35/44 (79.5)
Basket extraction using cholangioscopy‐guided retrieval basket	17/44 (38.4)
Electro‐hydraulic lithotripsy	1/44 (2.3)
Laser lithotripsy	1/44 (2.3)
Unsuccessful stone removal	1/45 (2.2)
Stone clearance validation by ERC after DSC (*n* = 46)**	
Confirmed complete stone clearance	42/46 (91.3)
Successful fluoroscopy‐free cannulation and complete stone clearance	38/46 (82.6)
Brief fluoroscopy used in cannulation and complete stone clearance by DSC guided only	4/46 (8.7)
Missed stone or incomplete removal by DSC but stone detected and removed during validation ERC	3/46 (6.5%)

Abbreviation: DSC, direct solitary cholangioscopy; ERC, endoscopic retrograde cholangiography.

* One patient with no stones found at the initial DSC and ERC validation procedure, and one patient with cannulation failure were excluded.

** One patient with no stones found at the initial DSC and ERC validation procedure was excluded.

Overall, in 46 cases in which cannulation was achieved, the mean cannulation time was 6.8 ± 6.4 min. In 43 patients undergoing successful standard cannulation technique, the mean cannulation time was 5.4 ± 3.6 min. In 3 patients achieving advanced techniques, the mean cannulation time was 26.9 ± 2.9 min.

### Stone removal at index DSC procedure

After successful cannulation in 46 patients, initial DSC evaluation did not show CBDS in one patient. In the remaining 45 procedures, the median number of CBDS determined by DSC was 1.0 (range 0–16). Papillary balloon dilation was performed in 13/45 (28.9%) patients using 8‐mm (2), 10‐mm (6), or 12‐mm (5) diameter balloons. Biliary sludge was seen in 15/45 (33.3%) cases.

Methods of stone removal, excluding one case in which no stones were observed during the index DSC, included single or combined use of an extraction balloon (35), mechanical lithotripsy (1), and cholangioscopy‐guided retrieval basket (17, in which seven relied solely on the cholangioscopy‐guided basket). Electro‐hydraulic lithotripsy and laser lithotripsy were each utilized in one patient. The mean number of CBDS removed was 1.9 ± 2.9 (range 0.0–16.0) for the cohort and 1.14 ± 0.38 for the seven patients with stone removal by the cholangioscopy‐guided basket. Biliary sludge or debris was removed in 19/45 (42.2%).

### Validation of stone clearance by ERC after DSC

One patient had no CBDS observed per DSC, nor in the validation ERC procedure. Of the remaining cases, in 42/46 (91.3%) stone clearance by cholangioscopy was successful in the index DSC procedure and subsequently confirmed in the validation ERC procedure, in 1/46 (2.2%) a CBDS was found in the salvage ERC procedure after fluoroscopy‐free cannulation failure, and in 3/46 (6.5%) incomplete stone clearance was noted. The incomplete stone clearance cases included a relatively large CBDS (10.3‐mm diameter) for which stone removal failed despite papillary balloon dilation (1), a patient in whom no CBDS was seen on cholangioscopy but was visualized by ERC (1), and a patient requiring mechanical lithotripsy with CBDS thought to be cleared, in whom the validation ERC found small stone fragments (1). All three patients with incomplete stone clearance in the index DSC procedure underwent successful stone removal with balloon extraction under fluoroscopy at the validation ERC procedure.

### Image quality, procedural duration, fluoroscopy time, and radiation exposure

During the DSC index procedure, cholangioscopic image quality was reported as excellent in 31/47 (66.0%), good in 14/47 (29.8%), fair in 1/47 (2.1%), and unable to visualize in 1/47 (2.1%) in which the single‐use cholangioscope malfunctioned and was replaced. The mean volume of saline used for irrigation during DSC was 71.3 ± 57.3 (range 10–220) ml. The mean procedural time was 26.7 ± 13.4 min and the mean total fluoroscopy time was 8.7 ± 42.6 min from duodenoscope intubation to the end of the DSC‐guided stone extraction procedure.

In those with brief fluoroscopy used in cannulation (4), the total mean radiation dose, mean DAP, and mean effective dose in the DSC procedure were 11.9 ± 12.9 mGy, 3147.9 ± 2937.3 Gy‐cm^2^, and 17.3 ± 20.0 μSv, respectively. In the ERC validation procedure, the total mean radiation dose, mean DAP, and mean effective dose were 21.4 ± 25.5 mGy, 2253.4 ± 2951.3 Gy‐cm^2^, and 30.6 ± 44.3 μSv. In the case of salvage ERC (1), the total mean radiation dose, mean DAP, and mean effective dose in the salvage ERC procedure were 27.7 mGy, 6.8 Gy‐cm^2,^ and 1.7 μSv.

### Initial experiences of DSC‐assisted stone removal among 12 endoscopists

See Supporting Information

### Serious adverse events through 30 days postprocedure

Through 30 days postprocedure, there was one SAE (1/47, 2.1%, 95% confidence interval 0.1–11.3), namely postprocedure pancreatitis, which resolved in 3 days. Reintervention was necessary for pancreatic stent removal in one patient who experienced cannulation failure. Four patients were lost to follow‐up, one after the 24‐h follow‐up visit and three after the 7‐day follow‐up visit.

## DISCUSSION

Our multicenter, multinational study of roll‐in cases demonstrates that DSC‐assisted stone removal is both feasible and safe when fluoroscopy and ERC are available if needed. We demonstrated the technique could be acquired within five cases by experts in ERCP and cholangioscopy.

The technique of DSC‐guided CBDS removal is effective and comparable with other modalities such as EUS‐assisted ERCP with CBDS removal (90%–95% vs. 85%).^14^, ^18^, ^20^ A prospective randomized trial^14^ demonstrated EUS‐assisted ERC as an effective modality to confirm stone clearance, but with significantly lower stone clearance rates compared to conventional ERC (85% vs. 100%; *p* = 0.002).^14^ The EUS‐based technique's main limitation was the inability to delineate the number of CBDS or differentiate bile duct stones from air bubbles after sphincterotomy.^21^ In contrast, DSC can accurately detect the number of stones and confirm CBDS clearance. EUS guidance is inefficient, requiring an exchange from echoendoscope for stone detection to duodenoscope for stone removal, then back to echoendoscope to confirm stone clearance, with repeated exchanges if additional retained stones are detected. In contrast, cholangioscopic guidance does not require an exchange of the duodenoscope. With the recent availability of cholangioscope‐compatible stone baskets, stone extraction is even more simplified and will not require the exchange to an extraction balloon, all under fluoroscopy‐free cholangioscopic control. In all seven patients where only cholangioscope‐compatible stone retrieval baskets were used in our study, stones, and debris were cleared successfully in the DSC procedure without exchanging for an extraction balloon.

In two prospective single‐center studies from the US (*n* = 40) and Thailand (*n* = 50), endoscopists with prior DSC experience had high rates of successful fluoroscopy‐free cannulation (100% vs. 98%, respectively) and fluoroscopy‐free complete stone clearance following DSC‐assisted stone removal (95% and 90%, respectively).^13^, ^18^ Compared to these studies^13^, ^18^ our roll‐in experience showed relatively lower rates of successful fluoroscopy‐free cannulation (89.4% vs. 98%–100%) and fluoroscopy‐free complete stone clearance as we limited the cannulation time and allowed brief fluoroscopy to assist cannulation. Our missed CBDS rate after DSC detected by the subsequent ERC was comparable to the previous studies, namely 5%–6%^13^ and 6.3%.^18^

The majority of successful fluoroscopy‐free CBDS removal in prior case series was performed by balloon extraction (97%–100%).^8^, ^9^, ^11^, ^12^, ^13^, ^18^ We demonstrated that the SpyGlass Retrieval Basket was useful for CBDS extraction in 17/47 (36.2%) study cases. The use of this novel device may save procedural time by eliminating the need to remove the cholangioscope, advance the balloon catheter for stone extraction, then reintroduce the cholangioscope to confirm stone clearance. Additionally, for large or impacted stones, electro‐hydraulic lithotripsy or laser lithotripsy can be easily performed under cholangioscopic guidance, avoiding the need for mechanical lithotripsy which is not amenable to a fluoroscopy‐free technique.

Despite various techniques for fluoroscopy‐free ERCP with non‐complex CBD stone removal (e.g., single‐operator cholangioscopy, EUS guidance^13^, ^14^, ^18^), a study of EUS‐assisted ERCP without fluoroscopy and our data found that 15% and 10% of patients respectively did not achieve fluoroscopy‐free ERCP with stone removal and required fluoroscopy guidance for either cannulation or stone removal.^14^ In clinical practice, percutaneous transhepatic biliary drainage under transabdominal US guidance at the bedside may be performed urgently if a dilated bile duct is seen on the US in patients who cannot undergo ERCP under fluoroscopy guidance after unsuccessful fluoroscopy‐free ERCP, such as those in intensive care. If patients with non‐complex biliary stones do not require urgent endoscopic treatments, they may be followed with conservative management while they are unable to receive radiation.

The main limitation of our roll‐in study is the lack of a standard ERC comparator group, which precluded the determination of whether DSC could reduce radiation exposure during CBDS clearance. Our ongoing clinical trial randomizes patients to either DSC or ERC. Three patients underwent treatment despite failing to meet eligibility criteria, that is, two patients received DSC‐guided stone clearance despite acute pancreatitis and a third treated patient showed five stones on baseline imaging, but more stones were found during the procedure. Each endoscopist was limited to five or fewer DSC roll‐in cases, leading to a small overall study size. Because all involved endoscopists were considered ERC and DSC experts, the described procedures should be limited to facilities with experts, and the learning curve observed in this study may not apply to the larger community of endoscopists. We are unable to discuss patient populations for whom DSC is most suitable because pregnant women were excluded from this study (to protect them from randomization to ERC with obligate radiation exposure), and we have limited information about participants’ baseline comorbid health conditions. Future studies of DSC should be designed to clarify feasibility, efficacy, and safety in specific patient populations of interest.

In conclusion, our preliminary data from the roll‐in phase of our multicenter international study indicates that fluoroscopy‐free DSC‐assisted stone can be adopted by experienced biliary endoscopists, and is safe and efficacious. It has the potential to minimize or eliminate radiation exposure to both patients and staff, though backup fluoroscopy and salvage ERC are sometimes needed. Additionally, the technique will be beneficial for pregnant patients and critically ill patients who cannot be safely transferred to the fluoroscopy unit. A randomized study comparing DSC‐assisted CBDS clearance to the standard ERC‐assisted CBDS clearance is warranted and is ongoing.

## CONFLICT OF INTEREST STATEMENT

The authors disclose the following:

Wiriyaporn Ridtitid: Research support from Boston Scientific Corporation

Rungsun Rerknimitr: Research support from Boston Scientific Corporation

Mohan Ramchandani: No disclosures

Sundeep Lakhtakia: No disclosures

Raj J Shah: Consultant for Boston Scientific Corporation and Olympus

Janak N Shah: Research support from Boston Scientific Corporation

Nirav Thosani: Consultant for Boston Scientific Corp, Pentax America, Ambu Inc; Speaker for Abbvie; Royalty from UpToDate; Creatorship Rights for ROSEAid Inc

Mahesh K Goenka: no disclosures

Guido Costamagna: Research grants from Cook Medical and Boston Scientific, Advisory Board Grant from Olympus

Mihir S Wagh: Consultant for Boston Scientific, Olympus, Medtronic, Fujifilm, ConMed and Incyte; Research support from Steris

Vincenzo Perri: No financial relationships with a commercial entity producing health‐care‐related products and/or services relevant to this article.

Joyce Peetermans: Full‐time employee of Boston Scientific Corporation

Pooja G Goswamy: Full‐time employee of Boston Scientific Corporation

Zoe Liu: Full‐time employee of Boston Scientific Corporation at the time of this study

Srey Yin: Full‐time employee of Boston Scientific Corporation at the time of this study

Subhas Banerjee: Boston Scientific: Consultant and research support; Ambys Medicines: consultant

## Supporting information



Supplemental Online Content

## References

[1] Samardzic J , Latic F , Kraljik D *et al*. Treatment of common bile duct stones–is the role of ERCP changed in era of minimally invasive surgery? Med Arh 2010; 64: 187–8. 20645517

[2] Obana T , Fujita N , Noda Y *et al*. Efficacy and safety of therapeutic ERCP for the elderly with choledocholithiasis: Comparison with younger patients. Intern Med 2010; 49: 1935–41. 20847495 10.2169/internalmedicine.49.3660

[3] Disario JA , Freeman ML , Bjorkman DJ *et al*. Endoscopic balloon dilation compared with sphincterotomy for extraction of bile duct stones. Gastroenterology 2004; 127: 1291–9. 15520997 10.1053/j.gastro.2004.07.017

[4] Dumonceau JM , Garcia‐Fernandez FJ , Verdun FR *et al*. Radiation protection in digestive endoscopy: European Society of Digestive Endoscopy (ESGE) guideline. Endoscopy 2012; 44: 408–21. 22438152 10.1055/s-0031-1291791

[5] Kwok K , Hasan N , Duloy A , Murad F , Nieto J , Day LW . American Society for Gastrointestinal Endoscopy radiation and fluoroscopy safety in GI endoscopy. Gastrointest Endosc 2021; 94: 685–97.e4. 34399965 10.1016/j.gie.2021.05.042

[6] ASGE Technology Comittee , Pedrosa MC , Farraye FA *et al*. Minimizing occupational hazards in endoscopy: Personal protective equipment, radiation safety, and ergonomics. Gastrointest Endosc 2010; 72: 227–35. 20537638 10.1016/j.gie.2010.01.071

[7] Trindade AJ , Brun A , Vamadevan AS *et al*. Use of bedside transabdominal US in facilitating emergent intensive care unit ERCP without fluoroscopy. Gastrointest Endosc 2015; 81: 1268–9. 25440689 10.1016/j.gie.2014.09.025

[8] Stavropoulos S , Larghi A , Verna E , Stevens P . Therapeutic endoscopic retrograde cholangiopancreatography without fluoroscopy in four critically ill patients using wire‐guided intraductal ultrasound. Endoscopy 2005; 37: 389–92. 15824953 10.1055/s-2005-861118

[9] Shelton J , Linder JD , Rivera‐Alsina ME , Tarnasky PR . Commitment, confirmation, and clearance: New techniques for nonradiation ERCP during pregnancy (with videos). Gastrointest Endosc 2008; 67: 364–8. 18226705 10.1016/j.gie.2007.09.036

[10] Vohra S , Holt EW , Bhat YM , Kane S , Shah JN , Binmoeller KF . Successful single‐session endosonography‐based endoscopic retrograde cholangiopancreatography without fluoroscopy in pregnant patients with suspected choledocholithiasis: A case series. J Hepatobiliary Pancreat Sci 2014; 21: 93–7. 23798477 10.1002/jhbp.7

[11] Ersoz G , Turan I , Tekin F , Ozutemiz O , Tekesin O . Nonradiation ERCP with endoscopic biliary sphincterotomy plus papillary balloon dilation for the treatment of choledocholithiasis during pregnancy. Surg Endosc 2016; 30: 222–8. 25840897 10.1007/s00464-015-4190-1

[12] Wu W , Faigel DO , Sun G , Yang Y . Non‐radiation endoscopic retrograde cholangiopancreatography in the management of choledocholithiasis during pregnancy. Dig Endosc 2014; 26: 691–700. 24861135 10.1111/den.12307

[13] Ridtitid W , Luangsukrerk T , Angsuwatcharakon P *et al*. Uncomplicated common bile duct stone removal guided by cholangioscopy versus conventional endoscopic retrograde cholangiopancreatography. Surg Endosc 2018; 32: 2704–12. 29101557 10.1007/s00464-017-5966-2

[14] Netinatsunton N , Sottisuporn J , Siriboon A *et al*. Prospective randomized trial of EUS‐assisted ERCP without fluoroscopy versus ERCP in common bile duct stone. Gastrointest Endosc 2017; 86: 1059–65. 28392365 10.1016/j.gie.2017.03.1539

[15] Shah JN , Bhat YM , Hamerski CM , Kane SD , Binmoeller KF . Feasibility of nonradiation EUS‐based ERCP in patients with uncomplicated choledocholithiasis (with video). Gastrointest Endosc 2016; 84: 764–9. 27040099 10.1016/j.gie.2016.03.1485

[16] Artifon EL , Kumar A , Eloubeidi MA *et al*. Prospective randomized trial of EUS versus ERCP‐guided common bile duct stone removal: An interim report (with video). Gastrointest Endosc 2009; 69: 238–43. 19185687 10.1016/j.gie.2008.05.020

[17] Park SY , Park CH , Lim SU *et al*. Intraductal US‐directed management of bile duct stones without radiocontrast cholangiography. Gastrointest Endosc 2015; 82: 939–43. 26232850 10.1016/j.gie.2015.06.015

[18] Barakat MT , Girotra M , Choudhary A , Huang RJ , Sethi S , Banerjee S . A prospective evaluation of radiation‐free direct solitary cholangioscopy for the management of choledocholithiasis. Gastrointest Endosc 2018; 87: 584–9.e1. 28797911 10.1016/j.gie.2017.07.042 PMC5801123

[19] ASGE Standards of Practice Committee , JL B , Abbas Fehmi SM *et al*. ASGE guideline on the role of endoscopy in the evaluation and management of choledocholithiasis. Gastrointest Endosc 2019; 89: 1075–105 e15. 30979521 10.1016/j.gie.2018.10.001 PMC8594622

[20] Ridtitid W , Piyachaturawat P , Teeratorn N , Angsuwatcharakon P , Kongkam P , Rerknimitr R . Single‐operator peroral cholangioscopy cystic duct cannulation for transpapillary gallbladder stent placement in patients with acute cholecystitis at moderate to high surgical risk (with videos). Gastrointest Endosc 2020; 92: 634–44. 32330504 10.1016/j.gie.2020.03.3866

[21] Palazzo L , Girollet PP , Salmeron M *et al*. Value of endoscopic ultrasonography in the diagnosis of common bile duct stones: Comparison with surgical exploration and ERCP. Gastrointest Endosc 1995; 42: 225–31. 7498687 10.1016/s0016-5107(95)70096-x

