# Supplementary material for: Endoscopic clearance of non‐complex biliary stones using fluoroscopy‐free direct solitary cholangioscopy: Initial multicenter experience
Source: DEN Open. 2023 Jun 1;4(1):e241. doi: 10.1002/deo2.241 (PMC10235796; doi:10.1002/deo2.241)
Supplement: Supplementary file 1 — Supplemental Online Content [file DEO2-4-e241-s001.docx]

**Supplemental Online Content**

Ridtitid W, Rerknimitr R, Ramchandani M, et al. Endoscopic clearance of non-complex biliary stones using fluoroscopy-free direct solitary cholangioscopy: initial multi-center experience. Digestive Endoscopy Open 2023.

**Supplement to Materials and methods**

*Fluoroscopy-free Direct Solitary Cholangioscopy Procedure*

*Fluoroscopy-free cannulation*

Prior non-invasive imaging was reviewed to determine the number and size of CBDS and the diameter of the bile duct. Patients received prophylactic antibiotics and rectal indomethacin before the procedure. Once the major papilla was identified, a sphincterotome with guidewire was used to cannulate the bile duct without fluoroscopy. The length of the guidewire was chosen by investigator discretion (not specified in the protocol). Following deep cannulation, aspiration was performed to evaluate for presence of bile, indicating successful biliary cannulation (**Video 1**). If clear fluid was aspirated, which might indicate inadvertent pancreatic duct cannulation, biliary duct cannulation was re-attempted. If fluoroscopy-free standard cannulation could not be achieved within 15 minutes, advanced cannulation techniques including double wire and/or trans-pancreatic septotomy cannulation techniques was performed as the next step. If no fluid was aspirated, or if uncertainty remained over which duct was accessed, a brief tap on the fluoroscopy pedal was allowed, to assess whether the guidewire appeared to be in the bile duct. If standard cannulation failed, and failure persisted using advanced cannulation techniques for an additional 15 minutes, this was considered a cannulation failure and the patient was converted to conventional ERC in which cannulation and stone removal were continued with fluoroscopy support. If brief fluoroscopy was used to confirm successful biliary cannulation but the DSC procedure for stone clearance was continued, then the procedure was reported as successful DSC CBDS clearance with brief use of fluoroscopy. Biliary sphincterotomy was performed following successful cannulation. Additional papillary large balloon dilation could be performed if the sphincterotomy was deemed inadequate.

*Definitions*

Non-complex biliary stones was defined as 5 or fewer stones in common bile or common hepatic duct with largest stone no larger than 10 mm in diameter. In case stones were not seen on baseline imaging, we performed ERCP in patients with high probability for choledocholithiasis based on the ASGE criteria^1^ and the diameter of the dilated CBD (bile duct diameter from >6 mm to 12 mm was used as a surrogate criterion for largest stone diameter). Acute pancreatitis at the time of screening was defined as abdominal pain and serum concentration of lipase (required) or amylase (optional) three or more times the upper limit of normal value.

Complete stone clearance was defined as complete CBDS clearance achieved at the index DSC procedure without using fluoroscopy and subsequently confirmed in a validation procedure by ERCP under fluoroscopy including an occlusion cholangiogram. Procedural duration was defined as time from duodenoscope-in to the completion of stone clearance procedure, not including the validation ERC procedure. Radiation exposure to the patient was evaluated via total radiation dose, Dose Area Product (DAP), and effective dose, from duodenoscope-in to completion of stone clearance, not including validation ERC procedure. All serious adverse events related to the DSC devices and/or procedure through 30 days post index procedure were evaluated, including pancreatitis, bleeding, cholangitis and perforation, as described according to the literature.^2,3^

*Exclusion Criteria*

Exclusion criteria were potentially vulnerable subjects, including but not limited to pregnant women, subjects in whom an endoscopic procedure was contraindicated, and biliary stones located in the intrahepatic ducts, cystic duct, or above a bile duct stricture. Also excluded were patients with a prior biliary sphincterotomy, septic cholangitis or acute pancreatitis at time of enrollment (see details in *Definitions* section), primary sclerosing cholangitis, surgically altered gastro-duodenal luminal anatomy other than prior Billroth I reconstruction, and/or coagulopathy or ongoing need for antic

*Secondary endpoints*

Secondary endpoints included: 1) biliary cannulation success at the index DSC procedure, 2) stone removal at the index DSC procedure, 3) ERC validation of stone clearance after DSC, 4) operator rating of image quality (rating scale:, good, fair, poor, unable to visualize [device deficiency form required], 5) duration of index procedure defined as time from duodenoscope in to completion of stone clearance, not including the validation DSC procedure in ERC arm and ERC procedure in DSC arm, 6) radiation exposure to the patient as measured by total fluoroscopy time, total radiation dose, Dose Area Product (DAP), and effective dose, from duodenoscope in to completion of stone clearance, not including the validation DSC procedure in ERC arm and ERC procedure in DSC arm, 7) serious adverse events (SAEs) including all deaths (related and unrelated), severity, onset, time to resolution related to the DSC devices and/or procedure and/or the ERC procedure through 30 days post procedure.

**Supplement to Results**

*Initial experiences of DSC-assisted stone removal among 12 endoscopists*

One to 3 roll-in cases were performed by 4 endoscopists, and 4 to 5 by 8 endoscopists. Mean duration of the DSC index procedure was 29±13 minutes for the first, second, and third roll-in cases (n=32) and was 29±17 minutes for the fourth and fifth roll-in cases (n=14) (p>0.05).

**Supplement to Discussion**

Compared to two prior studies,^4,5^ we reported a lower rate of SAEs with DSC-assisted CBDS removal (2.1% vs. 7.5-10%). The predominant SAE in the earlier studies was postprocedure pancreatitis, potentially resulting from unlimited duration of cannulation attempts with advanced techniques.^4,5^ Unlike our study, the earlier studies included patients with acute biliary pancreatitis and did not assess pre-enrollment serum amylase/lipase.^4,5^ Postprocedure cholangitis was not noted in our current study, possibly because we minimized the volume of irrigated water (mean volume =71.3 ml) and used intermittent suction and routine prophylactic antibiotics. Our postprocedural cholangitis rate was significantly lower than in previous studies using an ultra-slim cholangioscope (14%) (p=0.005).^6-8^ {Arya, 2004 #26}Although fluoroscopic assistance for cannulation was authorized, there was one cannulation failure. This implies that bedside CBDS clearance without fluoroscopy sometimes fails, and a back-up plan for fluoroscopy is necessary if biliary drainage is required. We recognize that due to the complexity and cost of the procedure in clinical practice, it may be impractical to reduce radiation exposure dose associated with failed DSC. The endoscopy staff should be prepared to implement standard radiation safety precautions if needed whenever DSC is attempted.

**References**

1. Committee ASoP, Buxbaum JL, Abbas Fehmi SM, et al. ASGE guideline on the role of endoscopy in the evaluation and management of choledocholithiasis. Gastrointest Endosc 2019;89:1075-105 e15.

2. Cotton PB, Lehman G, Vennes J, et al. Endoscopic sphincterotomy complications and their management: an attempt at consensus. Gastrointest Endosc 1991;37:383-93.

3. Banks PA, Bollen TL, Dervenis C, et al. Classification of acute pancreatitis--2012: revision of the Atlanta classification and definitions by international consensus. Gut 2013;62:102-11.

4. Ridtitid W, Luangsukrerk T, Angsuwatcharakon P, et al. Uncomplicated common bile duct stone removal guided by cholangioscopy versus conventional endoscopic retrograde cholangiopancreatography. Surg Endosc 2018;32:2704-12.

5. Barakat MT, Girotra M, Choudhary A, Huang RJ, Sethi S, Banerjee S. A prospective evaluation of radiation-free direct solitary cholangioscopy for the management of choledocholithiasis. Gastrointest Endosc 2018;87:584-9 e1.

6. Sethi A, Chen YK, Austin GL, et al. ERCP with cholangiopancreatoscopy may be associated with higher rates of complications than ERCP alone: a single-center experience. Gastrointest Endosc 2011;73:251-6.

7. Moon JH, Choi HJ. The role of direct peroral cholangioscopy using an ultraslim endoscope for biliary lesions: indications, limitations, and complications. Clin Endosc 2013;46:537-9.

8. Arya N, Nelles SE, Haber GB, Kim YI, Kortan PK. Electrohydraulic lithotripsy in 111 patients: a safe and effective therapy for difficult bile duct stones. Am J Gastroenterol 2004;99:2330-4.
